# Effect of *Rosa laevigata* on PM10-Induced Inflammatory Response of Human Lung Epithelial Cells

**DOI:** 10.1155/2020/2893609

**Published:** 2020-09-07

**Authors:** Hyun Min Ko, Seung-Han Choi, Yumi Kim, Eun-Jin An, Seung-Hyeon Lee, Kwanil Kim, Hee-Jae Jung, Hyeung-Jin Jang

**Affiliations:** ^1^College of Korean Medicine, Kyung Hee University, 26 Kyungheedae-ro, Dongdaemun-gu, Seoul 02447, Republic of Korea; ^2^Department of Science in Korean Medicine, Graduate School, Kyung Hee University, Seoul 02447, Republic of Korea; ^3^Department of Biological Science in Korean Medicine, College of Korean Medicine, Graduate School, Kyung Hee University, Seoul 02447, Republic of Korea; ^4^Department of Clinical Korean Medicine, Graduate School, Kyung Hee University, Seoul 02447, Republic of Korea; ^5^Division of Allergy, Immune and Respiratory System, Department of Internal Medicine, College of Korean Medicine, Kyung Hee University, 23 Kyungheedaero, Dongdaemun-gu, Seoul 02447, Republic of Korea

## Abstract

Particulate matter 10 (PM10) with a diameter of less than 10 mm causes inflammation and allergic reactions in the airways and lungs, which adversely affects asthmatic patients. In this study, we examined the anti-inflammatory effects of *Rosa laevigata* (RL), which has been previously investigated medicinally in Korea and China for the discovery of plant-derived anti-inflammatory agents with low side effects, using a PM10-induced lung inflammatory disease model. Using MTT assay, we confirmed that in A549 cells pretreated with RL, cytotoxicity induced by PM10 (100 *μ*g/mL) exposure was attenuated. In addition, western blotting revealed that RL suppressed the expression level of MAPK/NF-*κ*B pathways and its downstream signal, COX-2 in PM10-induced A549 cells. Moreover, real-time PCR demonstrated that RL downregulated the mRNA expression level of inflammatory cytokines (TNF-*α*, IL-1*β*, IL-6, IL-13, and IL-17) in PM10-induced A549 cells. Based on the results of this study, RL has been shown to relieve inflammation in the lungs due to PM10 exposure. Therefore, RL may be developed as a natural remedy for respiratory diseases caused by PM10 exposure.

## 1. Introduction

Asthma is one of the most common chronic immune diseases in humans and is characterized by chronic allergic inflammatory reactions in the airways, resulting in bronchoconstriction, vasodilation, activation of airway edema, and diseases occurring in all age groups from young to old [1, 2]. More than 300 million people worldwide have been diagnosed with asthma, and due to the continued increase in asthma prevalence, it is expected that the number of asthma patients will reach nearly 400 million by 2020 [3]. Air pollution by particulate matter 10 (PM10) is a risk factor for exacerbation of asthma [4, 5].

The main components of particulate matter caused by natural causes, such as volcanoes, forest fires, and dust storms, or by artificial causes, such as construction sites, mining operations, road dust tires, and brake wear, are organic ions (sulfates, nitrates, ammonium, sodium, potassium, calcium, magnesium, and chloride), organic chemicals, and polycyclic aromatic hydrocarbons (PAH) [6]. Among them, a substance with a particle diameter of 10 *μ*m or less is called PM10. Unlike larger particles that are filtered by the nasal and bronchial cilia, PM10 reaches the airways and alveoli directly, causing inflammation and irritating the bronchus, lowering lung function, increasing respiratory disease symptoms, and exacerbating asthma symptoms [7–9]. In fact, PM10 has been reported to increase hospitalization rates for asthma/chronic obstructive pulmonary disease (COPD) patients [10]. In recent years, the concentration of PM10 in the environment has been steadily decreasing in Korea, but is still higher than in other countries [11]. Due to rapid industrialization in Korea, the air quality has greatly deteriorated. In addition, health problems are increasing in the society as the number of days of dust storms in Asian countries, such as China and Mongolia, exceeds the air quality standard of PM10 even in spring, fall, and winter [12, 13]. Therefore, as public interest in PM10 increases, studies are being actively conducted to prevent or improve the adverse effects caused by PM10-induced inflammation and various diseases. Among the studies for the development of therapeutic agents, compounds derived from plants with few side effects are gaining attention as potential therapeutic agents.

*Rosa laevigata* (RL) is a plant belonging to the family Rosaceae that has long been used predominantly for medicinal purposes in Korea and China, and the fruits of RL are often used as tonic or painkillers [14]. A recent pharmacological study of RL has suggested that relief from various diseases including the function of flavonoids in relieving oxidative stress, inflammation, and apoptosis and total saponins in RL protect against liver disease caused by carbon tetrachloride [15–17]. However, the anti-inflammatory effect on epithelial cells in the lungs related to allergic asthma is the only study in our epidermal growth factor- (EGF-) induced inflammation model [18]. Therefore, in the present study, we investigated the mechanism through which PM10 causes inflammatory responses in pulmonary epithelial cells. In addition, we investigated the effectiveness and treatment mechanisms of RL in an inflammatory model induced by PM10.

## 2. Materials and Methods

### 2.1. Reagents

Fine dust (PM10-like) (European Reference Material ERM-CZ120) was purchased from Sigma-Aldrich (St. Louis, MO, USA). MTT (3-(4,5-dimethyl-2-thiazolyl)-2,5-diphenyl-[2H]-tetrazolium bromide) was purchased from Invitrogen (Waltham, MA, USA). Rabbit anti-NF-*κ*B p65, anti-COX-2, anti-Lamin B1, anti-phospho-ERK1/2, anti-ERK1/2, anti-phospho-JNK, anti-JNK, anti-phospho-p38, and anti-p38 were purchased from Cell Signaling Tech (CST, Beverly, MA, USA), and mouse anti-*β*-actin, goat anti-mouse IgG-HRP, and goat anti-rabbit IgG-HRP antibodies were purchased from Santa Cruz Biotechnology (Dallas, TX, USA).

### 2.2. Sample Preparation

RL extract preparation and UPLC/ESI-QTOF-MS analysis have been described in our previous study [18]. Briefly, RL was extracted with distilled water for 3 h at 100°C. The extract was filtered and evaporated using a rotary vacuum evaporator at 60°C. Finally, the extract was powdered by lyophilization at −80°C for 24 h.

### 2.3. Cell Line Culture

A549 cells were purchased from American Type Culture Collection (ATCC, MD, USA). The cells were cultured using Dulbecco's modified Eagle's medium mixed with 10% FBS (Gibco, NY, USA) and 1X antibiotic-antimycotic solution (ABAM, Corning Inc., NY, USA) at 37°C and 5% CO_2_ conditions.

### 2.4. MTT Assay

To assess the cytotoxic effect of PM10, A549 cells were seeded in a 96-well plate (1 × 10^4^ cells/well) and incubated for 24 h. After starvation, the cells were treated with PM10 at varying final concentrations of 25, 50, 100, 200, and 400 *μ*g/mL and incubated for 24 h. After removing media from each well, MTT solution (5 mg/mL) was added and reacted for 2 h [19]. Finally, 100 *μ*L of dimethyl sulfoxide (DMSO, Sigma-Aldrich, St. Louis, MO, USA) was added to dissolve formazan, and the absorbance was measured at 540 nm using a microplate reader (Bio-Rad, Hercules, CA, USA).

To evaluate the cell protective effect of RL, each concentration (15.625, 31.25, 62.5, 125, and 250 *μ*g/mL) was pretreated 1 h before PM10-induced cell damage. For PM10 treatment, PM10 (100 *μ*g/mL) was added and incubated for 24 h. MTT assay was then conducted following the method described above.

### 2.5. Western Blot Analysis

To assess the anti-inflammatory responses of RL, A549 cells were seeded in a 6-well plate (2 × 10^5^ cells/well) and incubated for 24 h. After starvation, the cells were pretreated with RL 125 and 250 *μ*g/mL. PM10 (100 *μ*g/mL) was then added to the cells and incubated for 24 h. Cells were harvested in fresh tubes and lysed using lysis buffer and samples were extracted using a nuclear and cytoplasmic extraction kit (NE-PER) (Thermo Scientific, Rockford, USA) [20]. Bio-Rad protein assay reagent (Bio-Rad, Hercules, CA, USA) was used to measure protein concentrations. Each sample was separated in a 10% SDS-PAGE gel at 120 V for 120 min and then electrotransferred to a nitrocellulose membrane for 75 min at 100 V. The membranes were blocked with 5% bovine serum albumin. The membranes were incubated with the primary antibody, COX-2 (1 : 1000), phospho-NF-*κ*B p65 (1 : 1000), NF-*κ*B p65 (1 : 1000), phospho-ERK (1 : 1000), ERK (1 : 1000), phospho-JNK (1 : 1000), JNK (1 : 1000), phospho-p38 (1 : 1000), p38 (1 : 1000), *β*-actin (1 : 5000), or Lamin B (1 : 3000), overnight at 4°C. Membranes were extensively washed and then incubated with one of the following secondary antibodies: goat anti-rabbit IgG-HRP and goat anti-mouse IgG-HRP. The protein expression levels were measured using an ImageQuant LAS 500 (GE Healthcare Life Sciences, NSW, Australia). The intensities of the specific bands were calculated using ImageJ software (NIH, NY, USA).

### 2.6. Total RNA Isolation and Real-Time Polymerase Chain Reaction (RT-PCR)

To analyze the transcription level of proinflammatory cytokines in A549 cells, we used real-time PCR. A549 cells were seeded in a 96-well plate (2 × 10^5^ cells/well) and incubated for 24 h. After starvation, the cells were pretreated with RL 125 and 250 *μ*g/mL. Next, PM10 (100 *μ*g/mL) was added and incubated for 24 h. The total RNA was isolated from each cell treatment using Hybrid-RTM (GeneAll, South Korea) [21, 22]. Total RNA (500 ng) was converted to cDNA using oligo (dT) at incubation conditions of 55°C for 60 min followed by 85°C for 5 min and then stored at 4°C until further use. Real-time quantitative PCR was performed using the Universal SYBR Green Master Mix (Applied Biosystems, USA). cDNA was amplified using the following conditions: 95°C for 15 min followed by 40 cycles at 95°C for 30 s, 59°C for 30 s, and 72°C for 30 s. Real-time PCR analysis was performed on an Applied Biosystems StepOne system (Applied Biosystems, USA) [23]. In this study, quantification based on the relative expression of a target gene versus GAPDH gene (2^-ΔΔCt^) was employed to determine the level of mRNA expression. The primer sequences used for the real-time PCR analysis are listed in Table 1.

### 2.7. Statistical Analysis

Statistical analysis of the data was performed using the GraphPad Prism 5 software package (GraphPad Software, San Diego, CA, USA). An unpaired test (one-tailed) was conducted to analyze all the data. Values less than 0.05 were considered significant. All data are presented as the mean ± SEM.

## 3. Results

### 3.1. COX-2 Expression Level and Cell Viability in A549 Cells Treated with PM10

**Figure 1:**
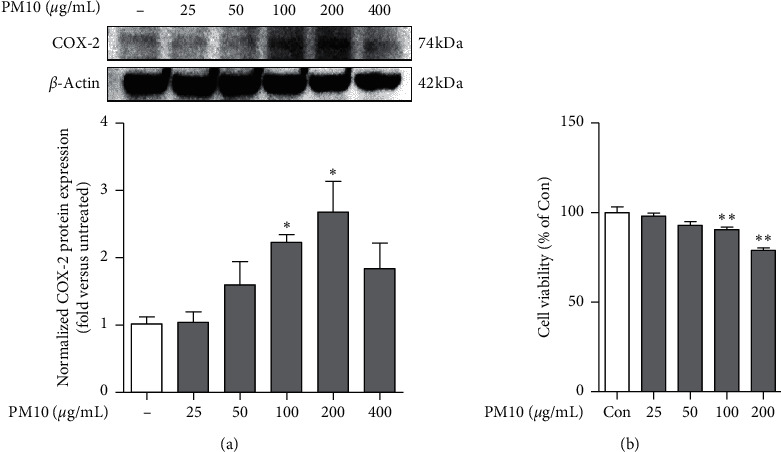
COX-2 expression level and cell viability in A549 cells treated with PM10. PM10 was treated for 24 h at the indicated concentrations. (a) COX-2 and actin proteins of A549 cells were confirmed by western blot analysis. (b) Cell viability of A549 cells was confirmed by MTT assay. Statistical significance was analyzed by unpaired *t*-test (one-tailed). Values are means ± SEM. ^*∗*^*P* < 0.05; ^*∗∗*^*P* < 0.01 compared to the control group.

COX-2 is a well-known proinflammatory gene that is overexpressed in terms of immune reactivity. Therefore, we used western blot analysis to examine PM10-induced inflammation in the A549 cells. When PM10 was added at 25–400 *μ*g/mL for 24 h in the A549 cell line, the expression level of COX-2 protein was as follows (Figure 1(a)): when PM10 was added at 100 and 200 *μ*g/mL concentrations, COX-2 expression level was significantly higher than the nontreated group. In addition, cell viability in the PM10-treated cells was confirmed using MTT assay (Figure 1(b)). When PM10 was added to the A549 cells at each concentration for 24 h, cell viability decreased in a concentration-dependent manner. In particular, there was a significant difference compared to the control at 100 and 200 *μ*g/mL. Therefore, considering these results, we selected the optimal concentration of PM10 as 100 *μ*g/mL for the subsequent studies.

### 3.2. Cell Protective Effects of RL on PM10-Treated A549 Cells

**Figure 2:**
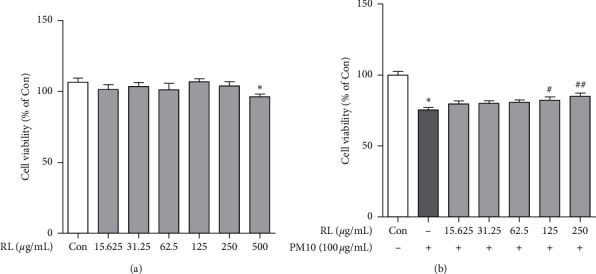
Cell protective effects of RL on PM10-treated A549 cells. (a) Cell viability of A549 cells treated with RL (15.625–500 *μ*g/mL) for 25 h. (b) RL (15.625–250 *μ*g/mL) was pretreated 1 h prior to PM10-induced cell damage in A549 cells and incubated for 24 h with PM10 (100 *μ*g/mL). Cell viability of A549 cells was confirmed by MTT assay. Statistical significance was analyzed by unpaired *t*-test (one-tailed). Values are means ± SEM. ^*∗*^*P* < 0.05 compared to the control group. ^#^*P* < 0.05; ^##^*P* < 0.01 compared to only PM10-treated group.

Before observing the anti-inflammatory effect of RL, we used MTT assay to analyze the cytotoxicity of RL. The cell viability of A549 cells treated with RL up to 500 *μ*g/mL for 25 h is shown in Figure 2(a). RL showed no toxicity to cells when treated at 250 *μ*g/mL, but cytotoxicity was observed at 500 *μ*g/mL. Therefore, we used RL concentrations of up to 250 *μ*g/mL for the subsequent studies. Based on the results in Figure 1, we observed the protective effect of RL against PM10-induced cytotoxicity. We found that the cell viability of A549 cells exposed to PM10 (100 *μ*g/mL) decreased by about 75% (Figure 2(b)). However, cell viability was increased when A549 cells were pretreated with 125 and 250 *μ*g/mL RL an hour before exposure to PM10. Therefore, the anti-inflammatory effect of RL was evaluated at 125 and 250 *μ*g/mL, which suppressed the PM10-induced decrease in cell viability.

### 3.3. Inhibitory Effects of RL on COX-2 Protein Expression in PM10-Treated A549 Cells

**Figure 3:**
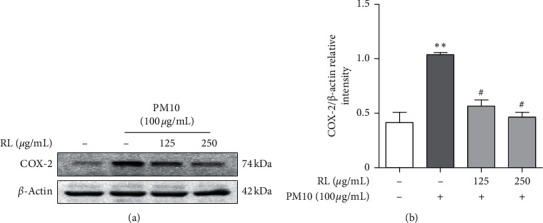
Inhibitory effects of RL on COX-2 protein expression in PM10-treated A549 cells. RL (125 and 250 *μ*g/mL) was pretreated 1 h prior to PM10-induced inflammation in cells and incubated for 24 h with PM10 (100 *μ*g/mL). Ratio of each protein was measured by ImageJ. Statistical significance was analyzed by unpaired *t*-test (one-tailed). Values are means ± SEM. ^*∗∗*^*P* < 0.01 compared to the control group. ^#^*P* < 0.05 compared to only PM10-treated group.

When A549 cells were exposed to 100 *μ*g/mL of PM10, COX-2 protein expression level increased (Figure 3). However, when cells were pretreated with RL at 125–250 *μ*g/mL for 1 h, COX-2 protein expression levels decreased in a dose-dependent manner compared to the PM10 induction group. Therefore, we further investigated the effect of RL on the upstream signal of COX-2 and proinflammatory cytokine expression.

### 3.4. Inhibitory Effects of RL on MAPK/NF-*κ*B Pathways in PM10-Treated A549 Cells

**Figure 4:**
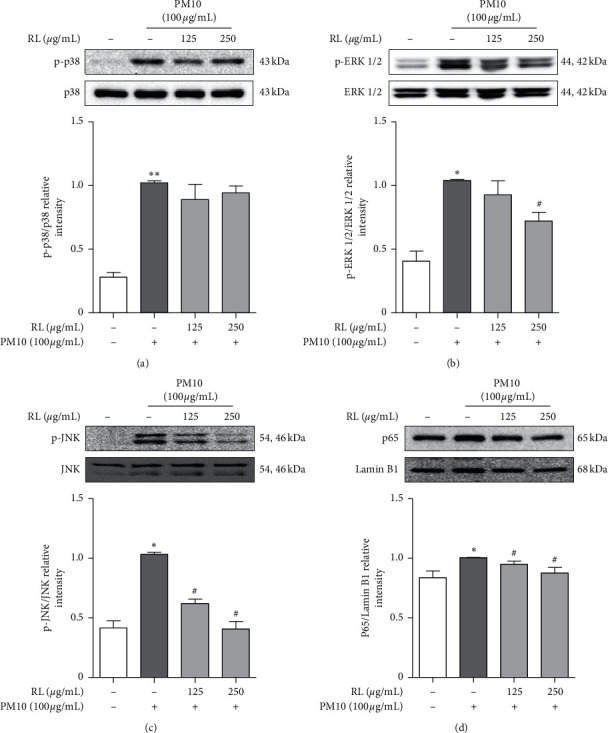
Inhibitory effects of RL on MAPK/NF-*κ*B pathways in PM10-treated A549 cells. RL (125 and 250 *μ*g/mL) was pretreated 1 h prior to PM10-induced inflammation in A549 cells and incubated for 24 h with PM10 (100 *μ*g/mL). (a)–(d) Inhibitory effects of RL on protein expression of p38, ERK 1/2, JNK, and NF-*κ*B p65 in PM10-treated A549 cells. Ratio of each protein was measured by ImageJ. Statistical significance was analyzed by unpaired *t*-test (one-tailed). Values are means ± SEM. ^*∗*^*P* < 0.05; ^*∗∗*^*P* < 0.01 compared to the control group. ^#^*P* < 0.05 compared to only PM10-treated group.

The mitogen-activated protein kinase (MAPK) and nuclear factor kappa-light-chain-enhancer of activated B cell (NF-*κ*B) pathway is activated by several stimuli and causes immune system dysfunction by expressing various inflammatory mediators. When A549 cells were treated with PM10 at a concentration of 100 *μ*g/mL, phosphorylation of c-Jun N-terminal kinase (JNK), extracellular signal-regulated kinase (ERK) 1/2, and p38, as well as translocation of NF-*κ*B p65 to the nucleus, was increased compared to the nontreated group (Figures 4(a)–4(d)). With RL pretreatment, phosphorylation of JNK and ERK decreased in a concentration-dependent manner. However, the phosphorylation of p38 tended to be somewhat suppressed, although there was no significant difference compared to the PM10 alone group. In addition, RL reduced the nuclear translocation of NF-*κ*B p65 in a concentration-dependent manner compared to the PM10 alone group. Consequently, RL mitigates PM10-induced inflammatory response through the MAPK/NF-*κ*B pathway.

### 3.5. Inhibitory Effects of RL on Proinflammatory Cytokine mRNA Expression in PM10-Treated A549 Cells

**Figure 5:**
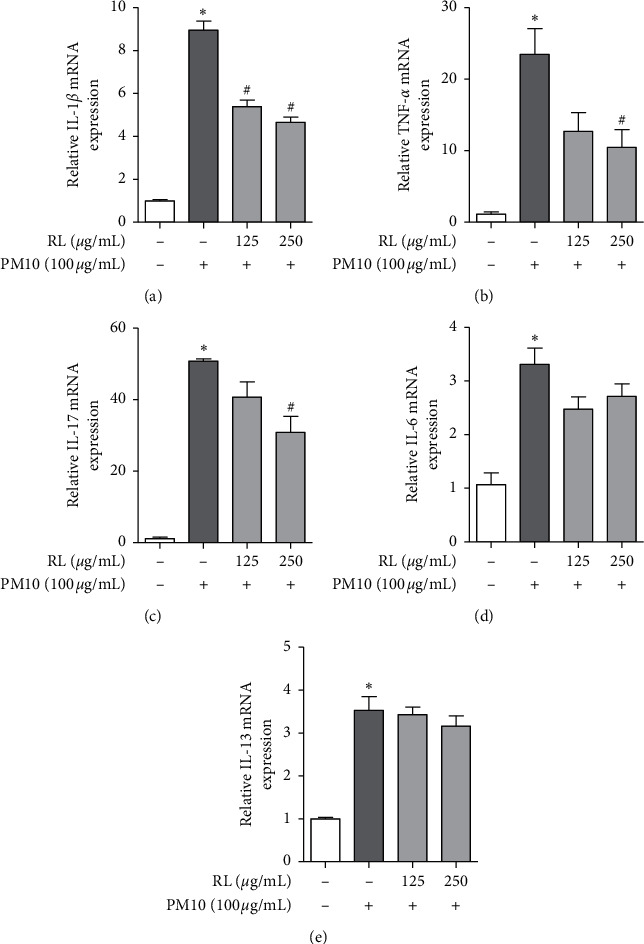
Inhibitory effects of RL on proinflammatory cytokine mRNA expression in PM10-treated A549 cells. RL (125 and 250 *μ*g/mL) was pretreated 1 h prior to PM10-induced inflammation in A549 cells and incubated for 24 h with PM10 (100 *μ*g/mL). (a)–(e) mRNA expressions of cytokine are detected by real-time PCR. Statistical significance was analyzed by unpaired *t*-test (one-tailed). Values are means ± SEM. ^*∗*^*P* < 0.05 compared to the control group. ^#^*P* < 0.05 compared to only PM10-treated group.

Using real-time PCR, we measured the mRNA expression levels of proinflammatory cytokines when A549 cells were exposed to PM10. The PM10-treated group had significantly higher mRNA expression levels of IL-6, IL-13, IL-17, IL-1*β*, and TNF-*α* than the nontreated group. However, the RL pretreatment group showed a concentration-dependent decrease in the mRNA expression of proinflammatory cytokines including IL-1*β*, TNF-*α*, and IL-17 (Figures 5(a)–5(c)). In addition, IL-6 and IL-13 did not show significant differences, but showed a tendency to decrease compared to the PM10 alone group (Figures 5(d) and 5(e)).

## 4. Discussion

PM10 is known to reach the bronchial tubes and lungs directly, aggravating asthma symptoms or increasing asthma rates. The PM10 used in this study was collected from road dust near a tunnel in Wislostrada, Poland, and is very similar to the fine dust collected in China [24]. According to previous studies, PM10 was used to induce increased mRNA expression of COX-2 protein and proinflammatory cytokines in HaCaT cells and RAW 264.7 cell lines [24]. Therefore, we investigated whether PM10 can induce inflammation by increasing the expression level of COX-2 protein in the A549 cell line. The COX enzyme, which converts arachidonic acid to prostaglandin H_2_, has two isoforms [25]. Among them, COX-2 is rapidly expressed during inflammation and is known to be involved in allergic asthma, arthritis, rheumatism, and various diseases [26, 27]. In this study, we treated A549 cells with PM10 at 25–400 *μ*g/mL for 24 h and found that the expression level of COX-2 was gradually increased in 100 and 200 *μ*g/mL treatment groups. In addition, it has been reported that ROS is generated by metals including iron, resulting in oxidative damage and inflammatory reactions, which subsequently results in cell death [28]. We examined cell viability when A549 cells were exposed to PM10 at concentrations of 25–200 *μ*g/mL and confirmed that the cells showed toxicity from 100 *μ*g/mL. Therefore, in this study, the exposure concentration of PM10 was set to 100 *μ*g/mL to analyze whether there is an anti-inflammatory effect of RL, which has been traditionally used as a medicinal plant in Asia, on PM10-treated alveolar epithelial cells.

When RL was pretreated at 15.625–250 *μ*g/mL concentration before PM10 was added to the A549 cells, we found that RL at 125 and 250 *μ*g/mL relieves cytotoxicity induced by PM10. In addition, cell viability increased up to 85.06%. Considering these results, RL may protect cells by mitigating the oxidative stress and inflammatory response induced by PM10. Therefore, to confirm the anti-inflammatory effects of RL, we set the treatment concentration of RL to 125 and 250 *μ*g/mL and proceeded to the next analysis.

COX-2 is a proinflammatory biomarker that produces PGE_2_, which mediates inflammation, pain, and fever [29]. Therefore, to confirm the anti-inflammatory effect of RL, we investigated whether the expression level of COX-2 following PM10 exposure was reduced by pretreatment with RL. After 24 h of exposure to PM10, the expression level of COX-2 was significantly increased; conversely, in RL-pretreated cells, PM10 expression was reduced in a dose-dependent manner. Therefore, RL may suppress the production of PGE_2_.

The MAPK pathway consists of ERK, JNK, and p38 kinase, which are known to play an important role in the proliferation of cells, controlling immune responses and activating inflammation related to the lungs. ERKs activated by growth factors, mitogen, and cytokines are mainly involved in cell mitosis, cell proliferation, and survival; when ERKs are overexpressed, cancer is induced [30]. JNK and p38 MAPK regulate the activity and expression of major inflammatory mediators including proteases and cytokines [31]. NF-*κ*B is a transcriptional regulator that plays a major role in regulating the expression of COX-2. Normally, it binds to I*κ*B and is present in the cytoplasm in an inactive state. However, when I*κ*B is phosphorylated and degraded by cytokine and LPS stimulation, NF-*κ*B is released into the nucleus and expression of various inflammatory factors, such as COX-2, cytokines, and chemokines, is induced [32].

In our study, when A549 cells were treated with PM10, MAPK phosphorylation and NF-*κ*B p65 nuclear translocation were activated. Pretreatment with RL inhibited the activation of MAPK phosphorylation by PM10 treatment and suppressed nuclear translocation of NF-*κ*B p65. Therefore, RL is expected to have a therapeutic effect by alleviating lung inflammation through this mechanism, even in asthma symptoms exacerbated by PM10.

Inflammation progresses through the action of proinflammatory cytokines, and a representative method of treating inflammatory diseases is to regulate the action of cytokines. IL-1*β* and TNF-*α* mediate inflammation by activating NF-*κ*B and AP-1. IL-6 induces the ERK pathway to regulate the differentiation and activation of T lymphocytes and is a cytokine that has recently been targeted for treating chronic inflammatory diseases such as asthma and rheumatoid arthritis [33]. IL-13 increases the number of lung eosinophils and airway hypersensitivity, exacerbating allergic inflammation [34]. Asthma has been believed to be caused primarily by increased Th2 cytokines and eosinophils, but recently, asthma induced by environmental factors, such as air pollution and viral infections, has been found to be associated with an increase in neutrophils [35, 36]. IL-17 is one of the major Th17 cytokines that play an important role in the development and progression of asthma, and its levels are elevated in the lungs of asthma patients. IL-17 can also activate NF-*κ*B and MAPK signals [37].

We confirmed that proinflammatory cytokines (TNF-*α*, IL-1*β*, IL-6, IL-13, and IL-17) in A549 cells treated with PM10 had significantly increased mRNA expression. Furthermore, RL has been shown to reduce these proinflammatory cytokines activated by PM10. Therefore, we speculate that RL can alleviate asthma symptoms caused by air pollution by inhibiting proinflammatory cytokines, which increases eosinophils and neutrophils.

RL we used in this study was a medicinal herb used in previous experiments, and the compounds related to anti-inflammatory effects such as quercetin and epicatechin were identified by LC-MS analysis [18]. In addition, RL contains flavonoids such as apigenin and kaempferide, as well as saponins such as ursolic acid, oleanolic acid, and *β*-sitosterol [38]. Quercetin has been reported to inhibit PGE2 biosynthesis by downregulating cytokine-induced COX-2/iNOS in A549 cells [39]. In addition, it has been confirmed that Pb-induced inflammation in the rat kidney is alleviated by the regulation of the MAPK/NF-*κ*B signaling pathway, and apigenin inhibits the mRNA expression of proinflammatory cytokines induced by phorbol-12-myristate-13-acetate in A549 cells [40, 41]. Moreover, ursolic acid inhibited T- and B-cell activation, proliferation, and cytokine secretion, as well as the phosphorylation of ERK and JNK [42]. Our findings on RL-derived components support the hypothesis that RL alleviates PM10-induced inflammation in the lungs.

## 5. Conclusions

In summary, we confirmed that in A549 cells pretreated with *R*. *laevigata*, cytotoxicity induced by PM10 (100 *μ*g/mL) exposure was attenuated. In addition, RL suppressed the expression level of MAPK/NF-*κ*B pathways and its downstream signal, COX-2 in PM10-induced A549 cells. Moreover, RL downregulated the mRNA expression level of inflammatory cytokines (TNF-*α*, IL-1*β*, IL-6, IL-13, and IL-17) in PM10-induced A549 cells. Thus, RL may be developed as a natural remedy for respiratory diseases caused by PM10 exposure.

## Figures and Tables

**Table 1:** Primer sequence for real-time quantitative PCR (qPCR) analysis.

Genes	Forward primer (5′-3′)	Reverse primer (5′-3′)
GAPDH	GCCACATCGCTCAGACACC	CCCAATACGACCAAATCCGT
TNF-*α*	GCAGGTCTACTTTGGGTCATTG	GCGTTTGGGAAGGTTGGA
IL-1*β*	TCAGCCAATCTTCATTGCTCAA	TGGCGAGCTCAGGTACTTCTG
IL-6	AGGGCTCTTCGGCAAATGTA	GAAGGAATGCCCATTAACAACAA
IL-13	CTGCAGTGCCATCGAGAAGA	GACCTTGTGCGGGCAGAA
IL-17	GGAACGTGGACTACCACATG	GCGCAGGACCAGGATCTCT

## Data Availability

All data generated or analyzed during this study are included within the article.
